# Swept Source Optical Coherence Tomography Angiography for Contact Lens-Related Corneal Vascularization

**DOI:** 10.1155/2016/9685297

**Published:** 2016-09-26

**Authors:** Marcus Ang, Yijun Cai, Anna C. S. Tan

**Affiliations:** ^1^Singapore National Eye Center, Singapore Eye Research Institute, Singapore; ^2^Ophthalmology and Visual Sciences Department, Duke-NUS, Singapore; ^3^Moorfields Eye Hospital NHS Foundation Trust, London, UK

## Abstract

*Purpose*. To describe a novel technique of adapting a swept-source optical coherence tomography angiography (OCTA) to image corneal vascularization.* Methods*. In this pilot cross-sectional study, we obtained 3 × 3 mm scans, where 100,000 A-scans are acquired per second with optical axial resolution of 8 *μ*m and lateral resolution of 20 *μ*m. This was performed with manual “XYZ” focus without the anterior segment lens, until the focus of the corneoscleral surface was clearly seen and the vessels of interest were in focus on the corresponding red-free image. En face scans were evaluated based on image quality score and repeatability.* Results*. We analyzed scans from 10 eyes (10 patients) with corneal vascularization secondary to contact lens use in 4 quadrants, with substantial repeatability of scans in all quadrants (mean image quality score 2.7 ± 0.7; *κ* = 0.75). There was no significant difference in image quality scores comparing quadrants (superior temporal: 2.9 ± 0.6, superior nasal: 2.8 ± 0.4, inferior temporal: 2.5 ± 0.9, and inferior nasal: 2.4 ± 1.0; *P* = 0.276) and able to differentiate deep and superficial corneal vascularization.* Conclusion*. This early clinical study suggests that the swept-source OCTA used may be useful for examining corneal vascularization, which may have potential for clinical applications such as detecting early limbal stem cell damage.

## 1. Introduction

Recent developments in optical coherence tomography (OCT) technology have now enabled clinicians to visualize vascular flow within the retina and choroid and around the optic disc [1, 2]. Optical coherence tomography angiography (OCTA) detects blood flow by analyzing signal decorrelation or phase deviations between ultra-high speed scans [3]. While OCTA may be potentially useful in assessing retinal or choroidal vessels, anterior segment vasculature imaging is still currently limited to slit-lamp photography or invasive angiography techniques, which expose patients to potential adverse reactions [4]. The first commercially available OCTA system (AngioVue, Optovue Inc., Fremont, CA, USA) uses spectral-domain technology, an 840 nm laser, and the split-spectrum amplitude-decorrelation algorithm (SSADA) to improve the signal-to-noise signal of flow detection [5]. Recently, a swept-source OCT system (Deep Range Imaging OCT, Topcon, Tokyo, Japan) has been developed for faster scans and deeper penetration. However, the OCTA functions in both of these systems were designed and optimized specifically for the retina, choroid, and optic disc vascular network. Therefore, in this proof of concept study we investigated the technique of adapting this OCTA device intended for retinal vessel imaging, modified to image corneal vascularization secondary to contact lens use, and discuss the limitations and potential benefits of this relatively new OCTA system.

## 2. Materials and Methods

In this pilot cross-sectional study, we analyzed 10 eyes of 10 subjects (5 male, 5 female; mean 24 ± 2 years of age) with evidence of corneal vascularization (daily contact lens wear, more than 5 years) but otherwise normal examination on slit-lamp evaluation at the Singapore National Eye Center with OCTA imaging from 1st to 30th of October 2015. Our study followed the principles of the Declaration of Helsinki, with ethics approval obtained from our local Institutional Review Board.

### 2.1. Imaging Technique

All scans were performed by a trained technician using the swept source DRI OCT (Triton, Topcon, Tokyo, Japan) which uses a wavelength-sweeping laser with a center wavelength of 1050 nm and a tuning range of approximately 100 nm. We used the “OCT Angiography” function to obtain 3 × 3 mm scans, where 100,000 A-scans are acquired per second with optical axial resolution of 8 *μ*m and lateral resolution of 20 *μ*m. This was performed without the anterior segment lens as the default focus of the system was designed for the retina and choroid. The eye tracking function was disabled and manual adjustments to the “XYZ” and focal length had to be made until two end-points were achieved: (1) the focus of the corneoscleral surface was clearly seen on the B-scan and (2) the vessels of interest were in focus on the red free image. En face OCT images were examined and correlated with the depth of corneal vascularization and scans from the cornea were taken in four quadrants to obtain 360 degrees of limbal scans.

### 2.2. Image Analysis

All scans were obtained with a minimum signal strength index of 50 and above. For the proof-of-concept phase of the study, all subjects had OCTA scans in four quadrants of the cornea limbus (superior temporal and nasal; inferior temporal and nasal) by a trained operator using the modified technique described. The quality of the scan images was assessed using the signal strength index; and image quality score was assessed using a recognized system that is 0 to 4 (0, no vessel discernible; 1, poor vessel delineation; 2, good vessel delineation; 3, very good vessel delineation; 4, excellent vessel delineation) on 2 scans per quadrant, performed by two independent masked assessors (MA, CY) [5]. We calculated the kappa coefficient (*κ*) value for the repeatability of scans using the image quality score, where *κ* ≤ 0.2 was considered slight, 0.21–0.40 weak, 0.41–0.6 moderate, 0.61–0.8 substantial, and 0.81–1.0 “almost perfect” in agreement.

## 3. Results

**Figure 1:**
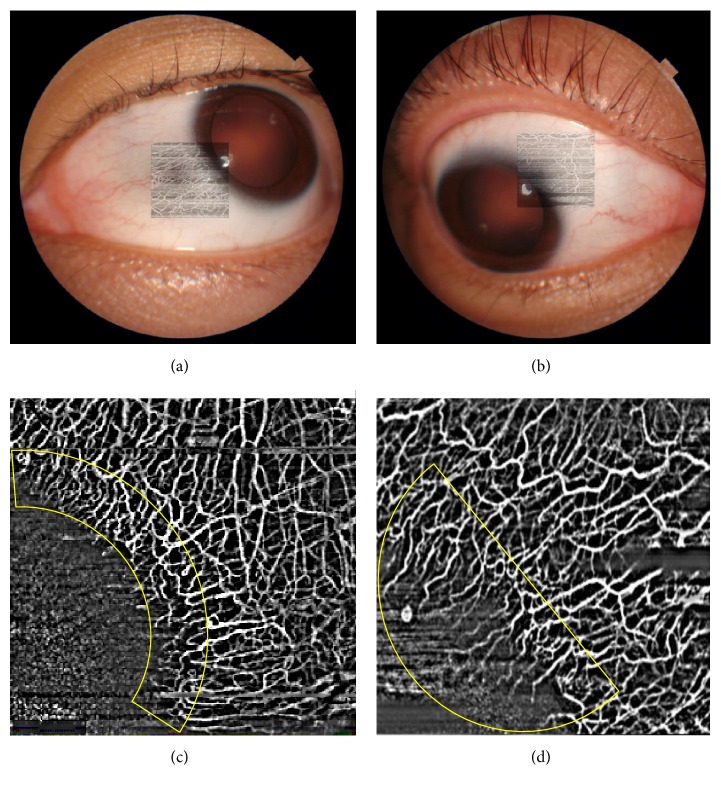
Examples of optical coherence tomography angiography (OCTA) images obtained from the swept-source optical coherence tomography system. (a) Anterior segment color photograph with OCTA image overlay is a unique feature of this system. (b) The OCTA scan image in combination with the slit-lamp photograph may be useful for localizing the pathology or evaluating the adjacent area. (c) Example of an OCTA scan that delineates normal vessels at the limbus after image processing. (d) Example of an OCTA scan that delineates abnormal corneal vascularization after image processing.

In this preliminary study, we analyzed scans from 10 eyes (4 scans per eye from each quadrant) with corneal vascularization. Overall we found substantial repeatability of scans in all quadrants in terms of image quality score (mean image quality score 2.7 ± 0.7; *κ* = 0.75). There was no significant difference in image quality scores comparing quadrants (superior temporal: 2.9 ± 0.6, superior nasal: 2.8 ± 0.4, inferior temporal: 2.5 ± 0.9, and inferior nasal: 2.4 ± 1.0; *P* = 0.276). However, we noted the highest percentage of poor image quality scores (score < 1) in the inferior nasal quadrant (20%) and inferior temporal quadrants (10%). We found that the swept-source OCTA system used here was able to image the anterior segment vasculature at various depths, with a feature to overlay the OCTA image over the color photograph, Figure 1.

## 4. Discussion

In this preliminary study, we describe a novel application of OCTA technology designed for retinal scans, to achieve noninvasive imaging of anterior segment vasculature by modifying the scan technique. As OCTA is still not widely used in the clinical setting, the purpose of this clinical pilot study was to describe a scan technique using this swept-source OCTA system and report its repeatability in terms of image quality. We had previously described the technique of adapting a commercially available spectral-domain OCTA system for the anterior segment [5]. The SSADA OCTA system obtains 3 to 6 mm scan cubes at a 70 kHz A-scan rate with lateral and axial resolutions at 15 *µ*m [6]. However, this technique has a relatively poor axial resolution (~15 *µ*m) due to signal averaging, which limits the identification of vessels with smaller caliber or diameters, still suffering from image distortions despite an in-built motion correction, and has a limited field of view [7].

The swept-source OCTA system used in this study has purported advantages of a better penetration of the deeper layers of the eye, a faster scanning speed (100 kHz A scan rate), and wider field of view. Specific to the macula and disc scans, the swept-source OCTA used here has a registration and tracking system allows for serial scans and monitoring, which is not currently available for the anterior segment. Our initial scans performed in this study may suggest that the scans provide better penetration and enhanced resolution of small vessels, but this requires direct comparative studies between OCTA systems used to scan the same eyes in future studies. Despite these potential benefits, our early experience with this system revealed that each scan still required 4-5 seconds, and the images also suffered from significant motion artefacts as seen in our illustrated figure.

Our observations from this early work suggest that OCTA may provide a promising noninvasive imaging alternative to FA or ICGA, both of which have been well-described for detecting areas of vascularization in the anterior segment [8, 9]. We recognize the limitations of our preliminary cross-sectional study, adapting the use of this novel OCTA system for the cornea and anterior segment. A large prospective study of various corneal pathologies with comparisons to other OCTA systems, slit-lamp photography, or ICGA would have been ideal to evaluate this OCTA technology [10]. Nevertheless, we present promising results that suggest this rapid, noninvasive OCTA system has the potential to develop further for imaging corneal vascularization, which may aid in further understanding of its role in various corneal, conjunctival, and scleral diseases in the future [11].
